# Metastasis Risk Assessment Using BAG2 Expression by Cancer-Associated Fibroblast and Tumor Cells in Patients with Breast Cancer

**DOI:** 10.3390/cancers13184654

**Published:** 2021-09-16

**Authors:** Chang-Ik Yoon, Sung-Gwe Ahn, Yoon-Jin Cha, Dooreh Kim, Soong-June Bae, Ji-Hyung Lee, Akira Ooshima, Kyung-Min Yang, Seok-Hee Park, Seong-Jin Kim, Joon Jeong

**Affiliations:** 1 Division of Breast Surgery, Department of Surgery, Seoul St Mary’s Hospital, College of Medicine, The Catholic University of Korea, Seoul 06591, Korea; fayn03@catholic.ac.kr (C.-I.Y.); 22100863@cmcnu.or.kr (D.K.); 2 Department of Surgery, Gangnam Severance Hospital, Yonsei University College of Medicine, Seoul 06273, Korea; asg2004@yuhs.ac (S.-G.A.); mission815815@yuhs.ac (S.-J.B.); 3 Institute for Breast Cancer Precision Medicine, Yonsei University College of Medicine, Seoul 06273, Korea; yooncha@yuhs.ac; 4 Department of Pathology, Gangnam Severance Hospital, Yonsei University College of Medicine, Seoul 06273, Korea; 5 Department of Biological Sciences, Sungkyunkwan University, Seobu-ro 2066, Jangan-gu, Suwon 16419, Gyeonggi-do, Korea; wlgud1990@skku.edu (J.-H.L.); parks@skku.edu (S.-H.P.); 6 GILO Institute, GILO Foundation, Seoul 06668, Korea; aooshima@gilo.or.kr (A.O.); yangkm@snu.ac.kr (K.-M.Y.); jasonsjkim@snu.ac.kr (S.-J.K.); 7 Medpacto Inc., Seocho-gu, Seoul 06668, Korea

**Keywords:** BAG2, breast cancer, cancer-associated fibroblast, metastasis

## Abstract

**Simple Summary:**

Cancer-associated fibroblasts (CAFs) promote tumor progression and play an important role in evading immune surveillance. The previous study showed that BAG2 could be elevated in cancer associated fibroblasts (CAFs). Here, we evaluated BAG2 expression of CAF and tumor cells and assessed metastasis risk in patients with breast cancer. We found that patients with either BAG2-high or BAG2(+) CAF had significantly worse distant metastasis-free survival than those with BAG2-double negative. Evaluation of BAG2 expression on both CAFs and tumor cells could be helpful to estimate the risk of metastasis in breast cancer.

**Abstract:**

Few studies have examined the role of BAG2 in malignancies. We investigated the prognostic value of BAG2-expression in cancer-associated fibroblasts (CAFs) and tumor cells in predicting metastasis-free survival in patients with breast cancer. Tissue-microarray was constructed using human breast cancer tissues obtained by surgical resection between 1992 and 2015. BAG2 expression was evaluated by immunohistochemistry in CAFs or the tumor cells. BAG2 expression in the CAFs and cytoplasm of tumor cells was classified as positive and negative, and low and high, respectively. BAG2-CAF was evaluated in 310 patients and was positive in 67 (21.6%) patients. Kaplan–Meier plots showed that distant metastasis-free survival (DMFS) was lesser in patients with BAG2(+) CAF than in patients with BAG2(−) CAF (*p* = 0.039). Additionally, we classified the 310 patients into two groups: 109 in either BAG2-high or BAG2(+) CAF and 201 in BAG2-low and BAG2(−) CAF. DMFS was significantly reduced in patients with either BAG2-high or BAG2(+) CAF than in the patients of the other group (*p* = 0.005). Multivariable analysis demonstrated that DMFS was prolonged in patients with BAG2(−) CAF or BAG2-low. Evaluation of BAG2 expression on both CAFs and tumor cells could help in determining the risk of metastasis in breast cancer.

## 1. Introduction

The Bcl-2 associated athanogene (BAG) family was first reported as a group of proteins that prevented cell death through their interactions with B-cell lymphoma 2 proteins (Bcl-2) [1,2]. The BAG domain of the BAG family directly interacts with the ATPase domain of heat shock protein 70 (Hsp70)/heat shock chaperone 70 (Hsc70) [3]. The BAG protein is known as a negative regulator of the chaperone-associated ubiquitin ligase C terminus of Hsc70-interacting protein (CHIP), which participates in ubiquitin-mediated proteasomal degradation of misfolded substrate proteins [4].

Among these, BAG2 is known as an Hsp70/Hsc70 molecular chaperone-interacting group protein [2]. The regulatory function of BAG2 via inhibition of CHIP activity has been reported to be involved in neurodegenerative diseases such as Parkinson disease and Alzheimer’s disease [4,5]. Unlike BAG1, which has been widely studied as a favorable prognostic marker in breast cancer [6], the role of BAG2 in malignancies has been examined only in a few studies. A previous study of cancer cell lines showed that BAG2 overexpression promoted the accumulation of mutant p53 in the nucleus and inhibited the degradation of mutant p53 through E3 ligase mouse double minute 2 homolog (a negative regulator of the p53 tumor suppressor) [7]. Furthermore, our group showed that BAG2 regulates the dual functions of cathepsin-B, facilitating the progression of triple-negative breast cancer [8]. 

Cancer-associated fibroblast (CAF) is a constituent of the tumor stroma, which influences tumor growth, invasion, metastasis, and evasion of immune responses. Tumor cells and stromal cells, which include CAFs, interact and co-evolve to create a suitable micro-environment for tumor growth [9]. In solid carcinomas, including breast cancer, the presence of abundant CAFs is correlated with worse survival outcomes [10]. A recent study showed that BAG2 expression is elevated in fibroblasts co-cultured with ovarian cancer cells, indicating that BAG2 could be elevated in CAFs and playing a role in stress response and cellular senescence pathways [11]. However, the effect of BAG2 expression in CAF on the prognosis of breast cancer patients remains unknown. 

Here, we evaluated the prognostic impact of BAG2 expression in CAF on the metastasis-free survival in breast cancer. Furthermore, we stratified the metastasis-free survival in these patients based on BAG2 expression in both CAFs and tumor cells.

## 2. Materials and Methods

### 2.1. Materials

Available formalin-fixed paraffin-embedded (FFPE) tumor samples were collected from the database of breast cancer patients treated between January 1992 and December 2015 at Gangnam Severance Hospital, Yonsei University Medical College, Seoul, Korea. BAG2 immunohistochemistry (IHC) expression was evaluated in tissue micro-array (TMA) blocks using these FFPEs. We included the tissue samples from 310 patients whose BAG2 expression was successfully evaluated in both tumor cytoplasm and the stroma using TMA slides. All these patients were diagnosed with stage I to stage III primary breast cancer. These patients were treated according to standard protocols. The patients’ clinical data included age at the time of surgery, histological grade, tumor size, lymph node status, estrogen receptor (ER) status, progesterone receptor (PR) status, human epidermal growth factor receptor-2 (HER2) status, lympho-vascular invasion (LVI), treatment modalities, events of distant metastasis, and death. Tumor grade was determined using the modified Scarff–Bloom–Richardson grading system. The study was conducted in accordance with good clinical practice guidelines and per the Declaration of Helsinki, and the protocol was approved by the institutional review board (IRB) (No. 3-2018-0067) of Gangnam Severance Hospital. The need for informed consent was waived under the approval of the IRB because of the retrospective design of our study.

### 2.2. Immunohistochemistry and Molecular Subtyping

As described in previous studies [12], 3 µm thick tissue sections were cut from the TMA block. After deparaffinization and rehydration using xylene and alcohol graded solutions, respectively, immunohistochemistry (IHC) was performed using Ventana Discovery XT Automated Slide Stainer (Ventana Medical System, Tucson, AZ, USA). Cell conditioning 1 buffer (citrate buffer, pH 6.0; Ventana Medical System) was used for antigen retrieval. Appropriate positive and negative controls were included. Staining of all the IHC markers was assessed using light microscopy. A cut-off value of 1% nuclear staining or more was considered positive for ER and PR [13]. HER2 staining was interpreted based on the 2018 American Society of Clinical Oncology/College of American Pathologists guidelines [14]. Only strong and circumferential membranous HER2 expression (3+) was considered positive, while 0 and 1+ HER2 staining was considered negative. Cases with equivocal HER2 expression (2+) were further evaluated for HER-2 gene amplification using silver in situ hybridization.

### 2.3. TMA Blocks and IHC Staining for BAG2 Expression Evaluation

**Figure 1:**
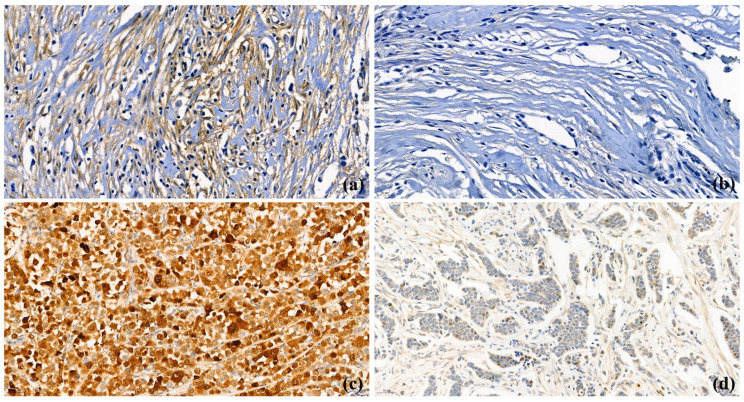
BAG2 expression in cancer-associated fibroblasts (CAFs) and cancer cells (400× magnification); (**a**) BAG2-positive CAFs in stroma; (**b**) BAG2-negative CAFs in stroma; (**c**) BAG2-high expression in the cytoplasm of primary breast cancer cells; (**d**) BAG2-low expression in the cytoplasm of primary breast cancer cells.

TMA paraffin blocks were constructed as previously described, using an Accu Max Array tissue-arraying instrument (Petagen Inc., Seoul, Korea) [12]. For IHC stating, each tissue microarray slide was stained with a BAG2-specific antibody (1:100, Rib monoclonal antibody, Abcam, Cambridge, UK), and counter-stained with hematoxylin. After staining, CAF- and cytoplasmic-BAG2 expression on each slide was scored by a pathologist (Yoon Jin Cha), using a light microscope (400× magnification). The results of IHC staining were scored as: negative (0), 1+, 2+, and 3+. As nuclear expression was rare and focal in most of the cases, the intensity of cytoplasm in the tumor cells and CAFs in the tumor-associated stroma were examined to grade the BAG2 expression (Figure 1). Normal luminal cells were used as internal control for staining intensity 2+. Weaker and strong nuclear staining as compared to the luminal cells were considered as 1+ and 3+, respectively. For BAG2 expression in CAFs, 1+ to 3+ were defined as positive (Figure 1a, BAG2-positive CAFs; Figure 1b, BAG2-negative CAFs). For cytoplasmic BAG2 expression of tumor cells, we considered negative and weak (1+) staining as low and moderate (2+) and strong (3+) expression as high, in accordance with the previous study (Figure 1c, BAG2-high expression in the cytoplasm of tumor cells, Figure 1d, BAG2-low expression in the cytoplasm of tumor cells) [8].

When analyzing the CAF- and tumor-BAG2 expression simultaneously, negative CAF- and low-cytoplasm-BAG2 expression were considered as double-negative, and all the other cases were classified as either BAG2(+) CAF or BAG2-high. IHC evaluation was carried out in a blinded manner, without any information regarding clinical parameters or outcomes.

### 2.4. Statistical Methods

Continuous variables were compared between the two groups using Student’s *t*-test or Mann–Whitney test. Categorical variables were compared using chi-squared test or Fisher’s exact test. Survival outcome and distant metastasis-free survival (DMFS) were depicted using the Kaplan–Meier method, and the two groups were compared using log-rank test. Univariate and multivariate Cox proportional hazard models were used to identify the factors related with DMFS. The variables used in the multivariate Cox proportional hazard model were those that showed statistical significance in the univariate analysis. DMFS was defined as the period from primary curative surgery to the date of systemic recurrence, death from any cause, or the last follow-up. To identify risk factors for metastasis, binary logistic regression model was used. Significant factors in univariable analyses were included in multivariable model.

SPSS version 24 (SPSS Inc., Chicago, IL, USA) was used for statistical analyses. Statistical significance was defined as *p*-value less than 0.05, and 95% confidence interval (CI) not including 1 was determined.

## 3. Results

### 3.1. Patients’ Characteristics According to BAG2 Expression in CAF

Breast cancer tissue samples from 310 patients were included in this study. Among these patients, 67 (21.6%) patients had positive BAG2 expression in CAFs and 243 (78.4%) patients had no BAG2 expression in their CAFs. Table 1 compares the clinical characteristics of both the groups (positive and negative BAG2-CAF).

Positive BAG2 expression in the CAFs was related with HER2-negative status and the treatment modalities of chemotherapy and radiotherapy. However, BAG2 positivity of CAFs was not related to larger tumor size, lymph node involvement, hormonal receptor status, or histological grade.

### 3.2. The Prognostic Influence of BAG2 Expression on Metastasis-Free Survival

At a median follow-up time of 111 months (6–325), 54 distant metastasis events occurred. First, we evaluated the prognostic influence of cytoplasmic BAG2 expression in tumor cells. The survival analysis suggested that DMFS tended to be lower in BAG2-positive patients than in BAG2-negative patients (Figure S1, *p* = 0.064). 

**Figure 2:**
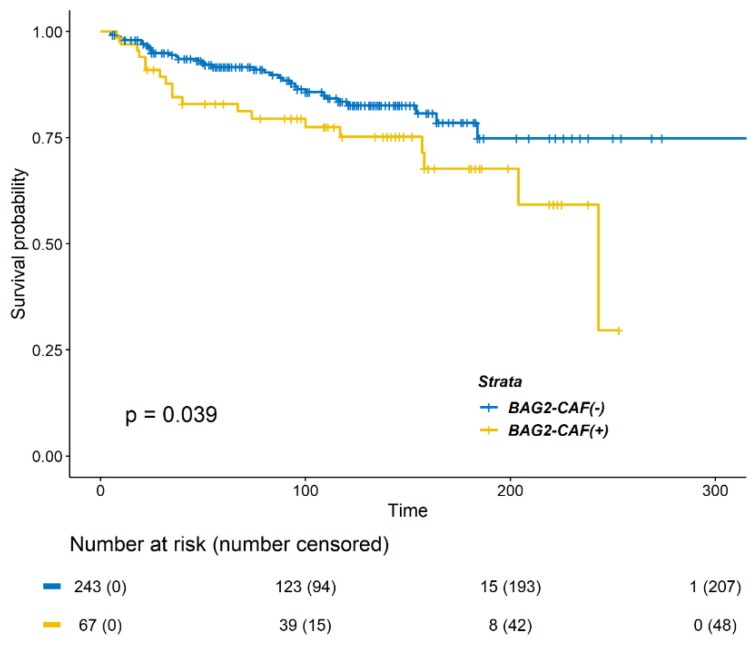
Kaplan–Meier survival curve of distant metastasis free survival (DMFS) according to BAG2 expression in cancer-associated fibroblasts (CAFs). Patients with positive BAG2 expression showed poorer DMFS (*p* = 0.0393, log-rank test).

Next, we evaluated DMFS according to BAG2 expression in CAFs. Positive BAG2 expression in CAF was significantly associated with decreased DMFS (Figure 2, *p =* 0.039 by log-rank test). Significant factors in univariate analysis for DMFS were as follows: age less than 40 years old at the time of surgery, tumor size larger than 2 cm, nodal metastasis, LVI, and positive BAG2 expression in CAF (Table 2). When adjusted for other factors, positive BAG2 in CAF was not a significant factor in reduced DMFS (Table 2, HR 1.584, 95% CIs 0.886–2.832, *p* = 0.121).

### 3.3. BAG2 Expression by the CAFs Combined with Cytoplasmic BAG2 Expression by the Tumor Cells

Of the 310 breast cancer patients, 10 (3.3%) patients had double positive for BAG2 expression by CAF and tumor cells, 99 (31.9%) had positive, and 201 (64.8%) patients had double negative BAG2 expression. DMFS significantly differed among these three groups (Figure S2; *p* = 0.018). Owing to the small number of patients in the double-positive group, we re-classified the patients into two groups: 109 (35.2%) in positive and 201 (64.8%) in double negative.

**Figure 3:**
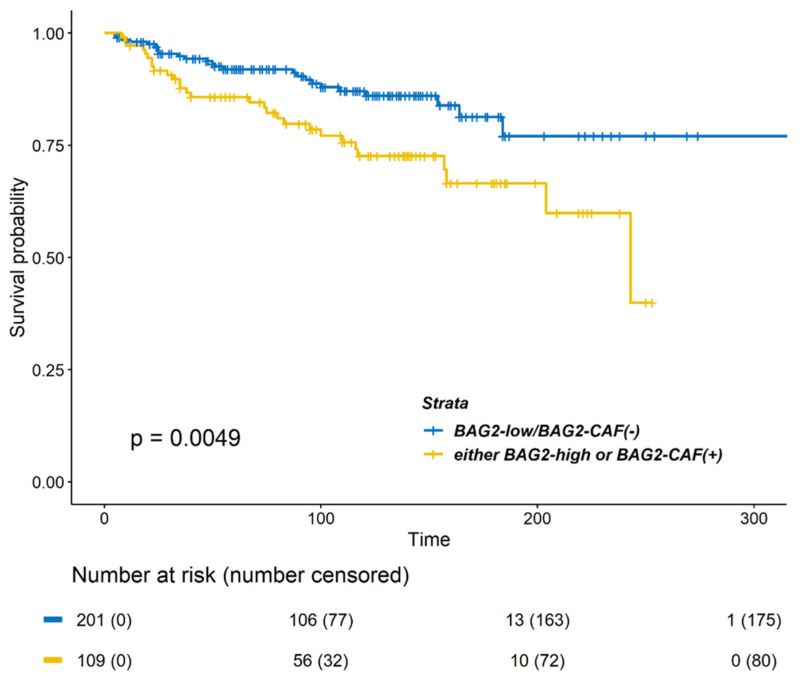
Kaplan–Meier survival curves of DMFS according to BAG2 expression in the cytoplasm and CAF. Patients with strong BAG2 expression (either high cytoplasm BAG2 or positive BAG2 in CAF) showed a poorer DMFS (*p* = 0.0049, log-rank test) as compared to patients with double-negative BAG2 expression (low cytoplasm BAG2 and negative BAG2 in CAF).

Clinical characteristics of both the groups (BAG2 expression positive and negative) are compared in Table 3. The group with either positive BAG2 in CAF- or tumor-cytoplasm was related only with a higher rate of receipt of chemotherapy and radiotherapy. DMFS differed significantly between the two groups (Figure 3; *p* = 0.0049). In the Cox proportional hazard model for DMFS, positive BAG2 expression was a significant factor in the multivariable analysis (Table 4, HR 1.764, 95% CIs 1.020–3.052, *p* = 0.042). 

To identify risk factors for metastasis per se, we conducted binary logistic regression analyses. In these analyses, positive BAG2 expression in cytoplasm and CAF was demonstrated to be a significant factor for distant metastasis (Table S1; HR 2.422, 95% CIs 1.260–4.658, *p* = 0.008).

## 4. Discussion

In this study, we found that BAG2 could be expressed in CAF and showed that tumors with BAG2+ CAF tend to have reduced DMFS in breast cancer. In addition, we demonstrated a negative prognostic value of BAG2 expression by either CAF or tumor cells for patients with breast cancer, in terms of DMFS. Our findings suggest that evaluation of BAG2 expression in the tumor stroma in addition to within the tumor cells could contribute to finer stratification of the metastatic risk in patients with breast cancer. As we already demonstrated that BAG2 expression in the tumor cytoplasm is associated with a reduced recurrence-free survival or breast cancer-specific survival, our findings have a novelty that the addition of BAG2 evaluation in tumor stroma could lead to better estimation of the risk of metastasis in breast cancer [8]. 

Interactions between the tumor and the CAFs play an important role in tumor progression in several solid cancers [15,16,17,18]. Across multiple studies, increasing CAF density or activated oncogenic signaling pathways in CAFs are found to be associated with poor prognosis in head and neck, oral, gastric, hepatocellular, and esophageal cancers [15,16,17,18]. Activated CAF may enhance metastasis, directly or indirectly, by releasing growth factors and cytokines to stimulate growth and epithelial-mesenchymal transition [19,20,21]. In breast cancer, CAFs-derived tenascin C and VEGFA are the key molecules involved in metastasis to the lung [22]. In addition, CAF-derived interleukin-32 promotes tumor cell invasion and metastasis by activating the p38 mitogen-activated protein kinase signaling [23]. Moreover, a recent study reported that the level of BAG2 protein is increased in fibroblasts that are co-cultured with ovarian cancer and inferred that BAG2 expression in CAF could be associated with tumor progression through multiple cellular processes [11]. From this viewpoint, our finding is noteworthy that elevated BAG2 expression in the tumor stroma could be a risk factor for metastasis in patients with breast cancer. 

Increasing evidence has implicated BAG2 in the pathogenesis of various diseases, including cancers and neurodegenerative diseases [4,5,24,25]. Particularly, the mechanisms through which BAG2 contributes to epithelial-mesenchymal transition by inhibiting the degradation of mutant p53 [7] or its interaction with microRNA-1180 [26] in cancer cells have been determined. Additionally, our previous study revealed that BAG2 stimulates distant metastasis by promoting the secretion of pro-cathepsin B in breast cancer [8]. These mechanistic studies support our results showing that high BAG2 expression is associated with frequent metastasis in patients with breast cancer. 

Interestingly, we previously noted that serum BAG2 levels were higher in patients with breast cancer than in healthy volunteers, suggesting that BAG2 in cancer patients could be secreted into blood [8]. Further studies are required to elucidate whether serum BAG2 level is associated with BAG2 expression in either tumor cells or CAFs.

Our study has some major limitations. Selection bias is inevitable owing to the retrospective nature of the study as we only used the available FFPEs that were recruited over a long time span. In addition, these subjects were treated with uncontrolled adjuvant treatments, which might have evolved gradually over the years. Furthermore, the sample size was small. As a result, the prognostic significance of conventional markers such as ER or subtypes was not reproducible in our study. In this context, the prognostic impact of BAG2 cytoplasmic expression by tumor cells on DMFS, which was underscored in our previous study, was not statistically significant in this study. Another limitation is that we did not use CAF-specific markers such as vimentin or alpha-smooth muscle actin [27]. Future study is warranted to address the relationship between CAF-related protein and BAG2 expression in breast cancer.

Despite these limitations, our study clearly revealed differential metastatic events according to BAG2 expression in patients with breast cancer. Additionally, our results suggest the prognostic value of measuring BAG2 expression in CAF along with that in the tumor cells of breast cancer. Thus, BAG2 may be a valuable target for preventing metastasis in patients with breast cancer.

## 5. Conclusions

We demonstrated that strong expression of BAG2 in either the tumor cells or CAFs is a significant risk factor for metastasis in patients with breast cancer. Further study is warranted to elucidate the role of BAG2 in the interaction between the cancer cells and the tumor stroma, which contributes to metastasis. Evaluation of BAG2 expression on both CAFs and tumor cells could lead to better evaluation of the risk of metastasis in patients with breast cancer.

## Figures and Tables

**Table 1:** Clinical characteristics according to BAG2 expression in cancer-associated fibroblast (CAF).

Variables	BAG2-Negative in CAF, *n* = 243 (%)	BAG2-Positive in CAF, *n* = 67 (%)	*p*-Value
Age (year, mean ± SD)	48.82 ± 10.94	47.36 ± 10.71	0.332
**ER**			0.501
Positive	163 (67.1)	42 (62.7)	
Negative	80 (32.9)	25 (37.3)	
**PR**			0.105
Positive	144 (59.3)	47 (70.1)	
Negative	99 (40.7)	20 (29.9)	
**HER2**			0.001
Positive	74 (30.5)	7 (10.4)	
Negative	151 (62.1)	55 (82.1)	
Missing	18 (7.4)	5 (7.5)	
**HG**			0.137
I, II	160 (65.8)	38 (56.7)	
III	68 (28.0)	25 (37.3)	
Missing	15 (6.2)	4 (6.0)	
**Tumor size**			0.665
≤2 cm	84 (34.6)	25 (37.3)	
>2 cm	156 (64.2)	41 (61.2)	
Missing	3 (1.2)	1 (1.5)	
**Lymph node metastasis**			0.723
Negative	121 (49.8)	32 (47.8)	
Positive	120 (49.4)	35 (52.2)	
Missing	2 (0.8)	0	
**LVI**			0.117
Negative	186 (76.5)	60 (89.6)	
Positive	38 (15.6)	6 (9.0)	
Missing	19 (7.8)	1 (1.5)	
**Breast surgery**			0.001
Wide excision	57 (23.5)	30 (44.8)	
Mastectomy	183 (75.3)	36 (53.7)	
Missing	3 (1.2)	1 (1.5)	
**Axilla surgery**			0.003
SLNB	62 (25.5)	6 (9.0)	
ALND	169 (69.5)	60 (89.6)	
Not done	9 (3.7)	0	
Missing	3 (1.2)	1 (1.5)	
**Chemotherapy**			0.024
Done	197 (81.1)	62 (92.5)	
Not done	41 (16.9)	4 (6.0)	
Unknown	5 (2.1)	1 (1.5)	
**Chemotherapy regimen (including duplicate)**			
Anthracycline	142 (72.1)	36 (58.1)	
Taxane	63 (32.0)	12 (19.4)	
Others	51 (25.9)	26 (41.9)	
**Radiotherapy**			0.001
Done	81 (33.3)	36 (53.7)	
Not done	159 (65.4)	28 (41.8)	
Unknown	3 (1.2)	3 (4.5)	
**Endocrine therapy**			0.460
Done	157 (64.6)	40 (59.7)	
Not done	86 (35.4)	27 (40.3)	
**Anti-estrogen regimen**			0.050
Tamoxifen	114 (72.6)	35 (87.5)	
Aromatase inhibitor	43 (27.4)	5 (12.5)	

SD, standard deviation; ER, estrogen receptor; PR, progesterone receptor; HER-2, human epidermal growth factor receptor-2; HG, histological grade; TNBC, triple negative breast cancer; LVI, lympho-vascular invasion; SLNB, sentinel lymph node biopsy; ALND, axillary lymph node dissection.

**Table 2:** Hazard ratios (HRs) and 95% confidence intervals (CIs) for distant metastasis-free survival (DMFS).

Variables	Univariate Analysis		Multivariate Analysis	
	HRs (95% CIs)	*p*-Value	HRs (95% CIs)	*p*-Value
**Age**		0.001		0.001
≤40	1		1	
>40	0.391 (0.229–0.669)		0.399 (0.231–0.691)	
**ER**		0.942		
Negative	1			
Positive	0.979 (0.556–1.725)			
**PR**		0.861		
Negative	1			
Positive	1.051 (0.604–1.827)			
**HER2**		0.886		
Negative	1			
Positive	1.047 (0.556–1.972)			
**HG**		0.752		
I, II	1			
III	0.954 (0.712–1.278)			
**Tumor size**		0.022		0.057
≤2 cm	1		1	
>2 cm	2.180 (1.121–4.236)		1.933 (0.981–3.807)	
**Lymph node metastasis**		0.002		0.013
Negative	1		1	
Positive	2.469 (1.387–4.392)		2.176 (1.179–4.017)	
**BAG2 expression in CAF**		0.042		0.121
Negative	1		1	
Positive	1.788 (1.021–3.133)		1.584 (0.886–2.832)	
**LVI**		0.020		0.272
Negative	1		1	
Positive	2.229 (1.133–4.385)		1.493 (0.731–3.051)	
**Chemotherapy**		0.192		
Not done	1			
Done	1.972 (0.711–5.466)			
**Radiotherapy**		0.106		
Not done	1			
Done	1.578 (0.908–2.741)			
**Endocrine therapy**		0.707		
Not done	1			
Done	0.900 (0.519–1.559)			

**Table 3:** Clinical characteristics according to BAG2 expression in cytoplasm and CAF.

Variables	Double-Negative BAG2 Expression in Cytoplasm and CAF, *n* = 201 (%)	Positive BAG2 Expression in Cytoplasm and CAF, *n* = 109 (%)	*p*-Value
Age (year, mean ± SD)	49.28 ± 11.10	47.07 ± 10.40	0.089
**ER**			0.786
Positive	134 (66.7)	71 (65.1)	
Negative	67 (33.3)	38 (34.9)	
**PR**			0.094
Positive	117 (58.2)	74 (67.9)	
Negative	84 (41.8)	35 (32.1)	
**HER2**			0.417
Positive	56 (27.9)	25 (22.9)	
Negative	132 (65.7)	74 (67.9)	
Missing	13 (6.5)	10 (9.2)	
**HG**			0.469
I, II	130 (64.7)	68 (62.4)	
III	57 (28.4)	36 (33.0)	
Missing	14 (7.0)	5 (4.6)	
**Tumor size**			0.825
≤2 cm	70 (34.8)	39 (35.8)	
>2 cm	129 (64.2)	68 (62.4)	
Missing	2 (1.0)	2 (1.8)	
**Lymph node metastasis**			0.383
Negative	103 (51.2)	50 (45.9)	
Positive	97 (48.3)	58 (53.2)	
Missing	1 (0.5)	1 (0.9)	
**LVI**			0.054
Negative	153 (76.1)	93 (85.3)	
Positive	34 (16.9)	10 (9.2)	
Missing	14 (7.0)	6 (5.5)	
**Breast surgery**			0.028
Wide excision	48 (23.9)	39 (35.8)	
Mastectomy	150 (74.6)	69 (63.3)	
Missing	3 (1.5)	1 (0.9)	
**Axilla surgery**			0.058
SLNB	46 (22.9)	22 (20.2)	
ALND	143 (71.1)	86 (78.9)	
Not done	9 (4.5)	0	
Missing	3 (1.5)	1 (0.9)	
**Chemotherapy**			0.018
Done	160 (79.6)	99 (90.8)	
Not done	36 (17.9)	9 (8.3)	
Unknown	5 (2.5)	1 (0.9)	
**Chemotherapy regimen (including duplicate)**			
Anthracycline	114 (71.3)	64 (64.6)	
Taxane	55 (34.4)	20 (20.2)	
Others	42 (26.3)	35 (35.4)	
**Radiotherapy**			0.023
Done	67 (33.3)	50 (45.9)	
Not done	131 (65.2)	56 (51.4)	
Unknown	3 (1.5)	3 (2.8)	
**Endocrine therapy**			0.575
Done	130 (64.7)	67 (61.5)	
Not done	71 (35.3)	42 (38.5)	
**Anti-estrogen regimen**			0.027
Tamoxifen	92 (70.8)	57 (85.1)	
Aromatase inhibitor	38 (29.2)	10 (14.9)	

**Table 4:** HRs and 95% CIs for DMFS at BAG2 expression in cytoplasm and CAF.

Variables	Univariate Analysis		Multivariate Analysis	
	HRs (95% CIs)	*p*-Value	HRs (95% CIs)	*p*-Value
**Age**		0.001		0.002
≤40	1		1	
>40	0.391 (0.229–0.669)		0.411 (0.237–0.713)	
**ER**		0.942		
Negative	1			
Positive	0.979 (0.556–1.725)			
**PR**		0.861		
Negative	1			
Positive	1.051 (0.604–1.827)			
**HER2**		0.886		
Negative	1			
Positive	1.047 (0.556–1.972)			
**HG**		0.752		
I, II	1			
III	0.954 (0.712–1.278)			
**Tumor size**		0.022		0.045
≤2 cm	1		1	
>2 cm	2.180 (1.121–4.236)		2.005 (1.016–3.959)	
**Lymph node metastasis**		0.002		0.018
Negative	1		1	
Positive	2.469 (1.387–4.392)		2.096 (1.135–3.874)	
**BAG2 expression**		0.006		0.042
Double Negative	1		1	
Either Positive	2.116 (1.239–3.614)		1.764 (1.020–3.052)	
**LVI**		0.020		0.191
Negative	1		1	
Positive	2.229 (1.133–4.385)		1.621 (0.786–3.344)	
**Chemotherapy**		0.192		
Not done	1			
Done	1.972 (0.711–5.466)			
**Radiotherapy**		0.106		
Not done	1			
Done	1.578 (0.908–2.741)			
**Endocrine therapy**		0.707		
Not done	1			
Done	0.900 (0.519–1.559)			

## Data Availability

All the data generated or analyzed during this study are included in this research article and its additional files.

## Supplementary Materials

The following are available online at https://www.mdpi.com/article/10.3390/cancers13184654/s1, Figure S1: Kaplan-Meier survival curve of distant metastasis free survival (DMFS) according to BAG2 expression in the tumor cytoplasm, Figure S2: Kaplan-Meier survival curves of DMFS according to BAG2 expression in the cytoplasm and CAF, Table S1: Hazard ratios (HRs) and 95% confidence intervals (CIs) for distant metastasis-free survival (DMFS) using binary logistic regression analysis.
