# Supplementary material for: Metastasis Risk Assessment Using BAG2 Expression by Cancer-Associated Fibroblast and Tumor Cells in Patients with Breast Cancer
Source: Cancers (Basel). 2021 Sep 16;13(18):4654. doi: 10.3390/cancers13184654 (PMC8470501; doi:10.3390/cancers13184654)
Supplement: Supplementary file 1 [file cancers-13-04654-s001.zip › cancers-1367132-supplementary.pdf]

cancers

MDPI

Supplementary Material: Metastasis Risk Assessment Using BAG2 Expression by Cancer-Associated Fibroblast and Tumor Cells in Patients with Breast Cancer

Chang-Ik Yoon, Sung-Gwe Ahn, Yoon-Jin Cha, Dooreh Kim, Soong-June Bae, Ji-Hyung Lee, Akira Ooshima, Kyung-Min Yang, Seok-Hee Park, Seong-Jin Kim and Joon Jeong

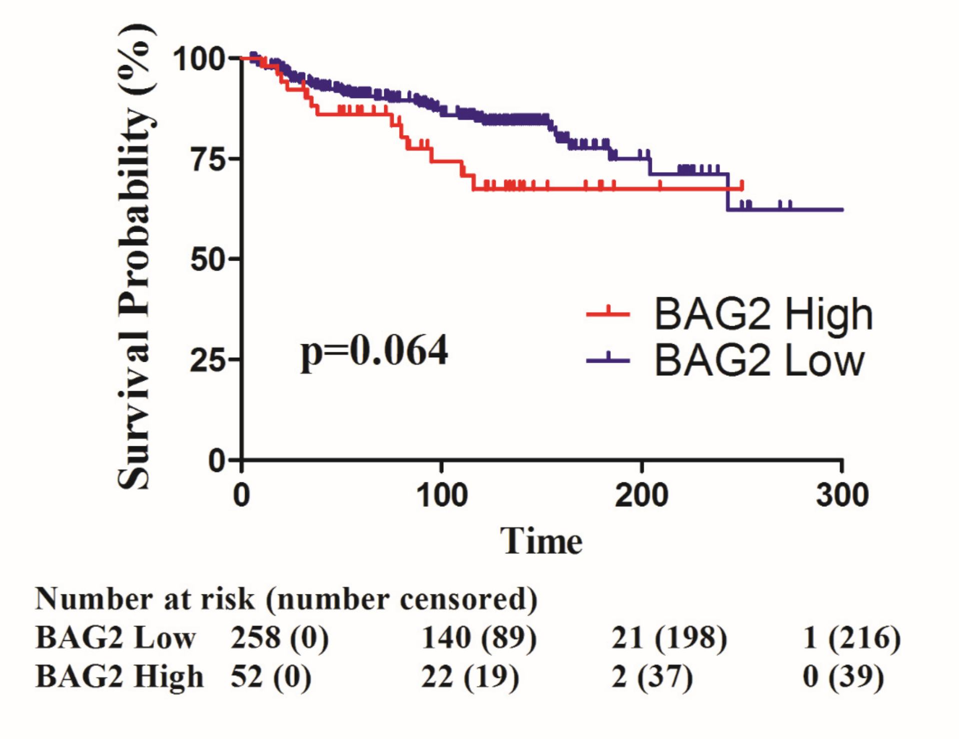

Figure S1. Kaplan-Meier survival curve of distant metastasis free survival (DMFS) according to BAG2 expression in the tumor cytoplasm. The DMFS did not differ by BAG2 expression in tumor cytoplasm significantly (p = 0.064, the log-rank test).

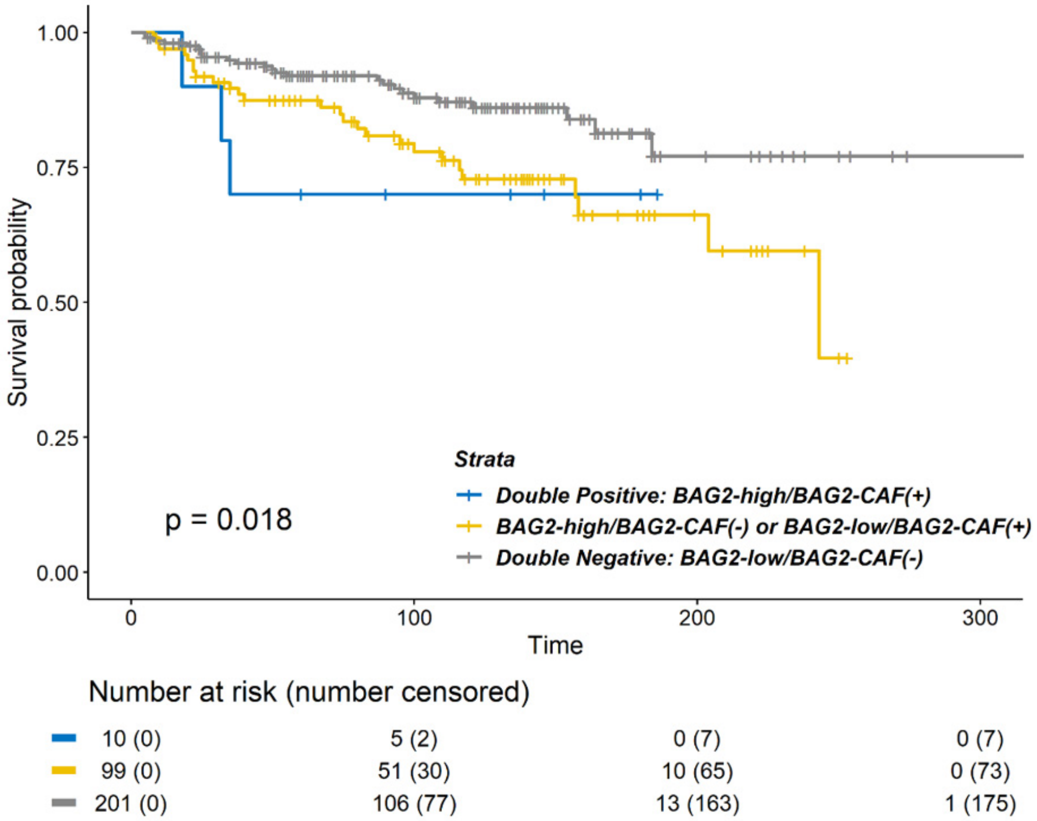

Figure S2. Kaplan-Meier survival curves of DMFS according to BAG2 expression in the cytoplasm and CAF. DMFS significantly differed among the three groups, which included 10 patients (3.3%) with double positive for BAG2 expression by CAF and tumor cells, 99 (31.9%) with either positive, and 201 (64.8%) patients with double-negative ($p = 0.018$, log-rank test).

Table S1. Hazard ratios (HRs) and 95% confidence intervals (CIs) for distant metastasis-free survival (DMFS) using binary logistic regression analysis.

Variables	Univariate Analysis		Multivariate Analysis	
	**HRs (95% CIs)**	***p* Value**	**HRs (95% CIs)**	***p* Value**
**Age**		<0.001		<0.001
≤40	1		1	
>40	0.282 (0.152–0.522)		0.288 (0.147–0.564)	
**ER**		0.927		
Negative	1			
Positive	1.030 (0.553–1.918)			
**PR**		0.822		
Negative	1			
Positive	1.072 (0.584–1.967)			
**HER2**		0.633		
Negative	1			
Positive	0.845 (0.424–1.685)			
**HG**		0.684		
I, II	1			
III	0.875 (0.459–1.666)			
**Tumor size**		0.015		0.008
≤2 cm	1		1	
>2 cm	2.414 (1.186–4.912)		2.993 (1.332–6.721)	
**Lymph node metastasis**		0.004		0.074
Negative	1		1	

Positive	2.508 (1.343–4.686)		1.860 (0.942–3.673)	
**BAG2 expression in cytoplasm and CAF**		0.002		0.008
Double-negative	1		1	
Positive	2.552 (1.405–4.634)		2.422 (1.260–4.658)	
**LVI**		0.188		
Negative	1			
Positive	1.667 (0.779–3.565)			
**Chemotherapy**		0.101		
Not done	1			
Done	2.452 (0.839–7.164)			
**Radiotherapy**		0.022		0.300
Not done	1		1	
Done	2.038 (1.110–3.739)		1.426 (0.729–2.790)	
**Endocrine therapy**		0.682		
Not done	1			
Done	0.882 (0.482–1.612)			
